# Health Impacts of Climate Change in Vanuatu: An Assessment and Adaptation Action Plan

**DOI:** 10.5539/gjhs.v5n3p42

**Published:** 2013-01-30

**Authors:** Jeffery T Spickett, Dianne Katscherian, Lachlan McIver

**Affiliations:** 1 Faculty of Health Sciences, School of Public Health and Curtin Innovation Research Institute (CHIRI), Curtin University, Perth, Western Australia, Australia; 2 WHO South Pacific Office, Suva, Republic of Fiji

**Keywords:** health impact assessment, climate change, adaptation, Vanuatu

## Abstract

Climate change is one of the greatest global challenges and Pacific island countries are particularly vulnerable due to, among other factors, their geography, demography and level of economic development.

A Health Impact Assessment (HIA) framework was used as a basis for the consideration of the potential health impacts of changes in the climate on the population of Vanuatu, to assess the risks and propose a range of potential adaptive responses appropriate for Vanuatu. The HIA process involved the participation of a broad range of stakeholders including expert sector representatives in the areas of bio-physical, socio-economic, infrastructure, environmental diseases and food, who provided informed comment and input into the understanding of the potential health impacts and development of adaptation strategies.

The risk associated with each of these impacts was assessed with the application of a qualitative process that considered both the consequences and the likelihood of each of the potential health impacts occurring. Potential adaptation strategies and actions were developed which could be used to mitigate the identified health impacts and provide responses which could be used by the various sectors in Vanuatu to contribute to future decision making processes associated with the health impacts of climate change.

## 1. Introduction

That climate change is already having an impact on the global burden of morbidity and mortality has been stated in the Intergovernmental Panel on Climate Change (IPCC) 4^th^ Assessment report, which also indicates that these effects are likely to increase all around the globe (IPCC, 2007). The nature and magnitude of climate change will determine the extent and nature of future health impacts, so it is crucial that strategies to mitigate climate change are widely implemented. However, irrespective of the implementation of mitigation measures, health impacts from climate change will ensue and therefore it is very important that adaptation measures are developed and implemented to ensure that adverse impacts are minimal (Cambell-Lendrum et al., 2006).

Although the changing climate is a worldwide issue, it will not be experienced uniformly across the world and many local and regional adaptation measures will need to be developed and implemented. It is crucial for the health of the community that adaptation strategies are implemented where the adverse health impacts that pose the greatest potential risk, and/or where the benefits to health can be maximised.

Understanding the relationship between climate variability, the environment and human health can enable us with some uncertainty, to predict the likely and plausible climate change-attributable impacts on health, and thus plan effective adaptation strategies (WHO, 2003a). Health impact pathways from climate change were first articulated by a special WHO Working Group in 1990; these pathways can occur as a result of direct or indirect exposures (WHO, 1990). Direct exposures refer to the immediate health impacts that can occur as a direct result of a climate variable, for example heat waves, fires, floods whereas indirect exposures occur when climate change affects various environmental parameters such as air, water, food quality, food production and disease vectors, or social parameters such as changes to population distribution and economic variables (IPCC, 2007). Pathways between changes in the climate and the subsequent impact on health for indirect exposures typically include a number of steps, many of which are not the responsibility of the health sector and call for a cross-sectoral, collaborative approach.

Potential points of vulnerability can occur at different steps in the health impact pathway and each step can present an opportunity for adaptation. Vulnerability can be considered as the degree to which a system is susceptible to, or unable to cope with, the adverse effects of climate change (IPCC, 2001) and is a function of three major factors; exposure to climate factors, sensitivity to change, and adaptive capacity. Exposure to climate factors depends on location and activities undertaken; sensitivity relates to the way the individual, community or system responds to climate change. Adaptive capacity is the general ability of institutions, systems and individuals to adjust to changes that occur as a result of climate change and the ability to take advantage of opportunities and to cope with the consequences. Many adaptation strategies will be designed to increase our capacity to adapt to the effects of climate change.

The involvement of a range of sectors in planning and implementing mitigation and adaptation strategies is important to optimise our responses to the various risk factors that determine our overall vulnerability. A comprehensive assessment of vulnerability is particularly important, as the main benefits from adaptation measures will occur when they focus on critical points in the pathway and/or at vulnerable sectors of the population.

## 2. Climate Change and Health in the Pacific

In 2009, the Health Ministers of the Pacific island countries held their biennial meeting in Madang, Papua New Guinea and included discussion on the impacts of climate change on health. The Ministers recognised that Pacific island countries are particularly vulnerable to climate change and identified several high-priority, climate-sensitive health risks common throughout the region (WHO, 2009). This initiative followed the dissemination of a pivotal WHO Regional Framework for Action document in 2008, which laid out key guidelines and core responsibilities for the health sector to protect communities from the health impacts of climate change in the Asia Pacific region (WHO, 2008).

In Vanuatu, as far back as 1999, in the country’s Initial National Communication (INC) to the United Nations Framework Convention on Climate Change (UNFCCC) consideration was given to the potential for climate change-attributable health impacts to occur (INC, 1999). More recently, the health impacts of climate change in Vanuatu were outlined in the National Adaptation Programme of Action (NAPA, 2007). In 2010, the Vanuatu Ministry of Health (MoH) commenced a twelve-month project, supported by the World Health Organization (WHO) South Pacific, aimed at improving the understanding of the relationship between climate and health in Vanuatu and to develop adaptation strategies related to climate change and health. This research was undertaken to identify potential risks to health, to evaluate the risks to determine their relative priority and then to develop potential adaptation strategies to minimise the impacts on *ni-Vanuatu* communities.

## 3. Assessment of Climate Change Health Impacts

Health Impact Assessment (HIA) is a tool developed to consider potential health issues during planning stages of proposals using established systematic mechanisms to demonstrate factors that could affect health and to consider potential management options in response. HIA is commonly defined as “a combination of procedures, methods and tools by which a policy, program or project may be judged as to its potential effects on the health of a population, and the distribution of those effects within the population” (WHO 1999). HIA is an evidence-based process that aims to identify and examine both the positive and negative health impacts of activities and provide decision makers with information about how the activity may affect the health of people.

The HIA framework follows the format of:

- Screening
- Scoping
- Profiling
- Risk assessment
- Risk management
- Decision making
- Evaluation

HIA has mainly been used for the assessments of projects or developments. However it has been identified by the World Health Organisation and others (Brown et al., 2011; Nelson, 2003; WHO, 2003b) that the HIA process provides an appropriate methodology by which the potential impacts of climate change could be initially assessed to support decision making, especially since it considers health equity (Patz et al., 2008). We developed an HIA framework that provided for the prediction of potential impacts based on a single possible scenario of future climatic conditions and biophysical changes in Western Australia (Spickett et al., 2011). The methodology was used as the basis for developing and implementing a process to develop potential adaptation strategies for health impacts from climate change in Vanuatu.

The health and well-being of the community is dependent on the activities of a range of private and public sectors including sectors such as environment, transport, energy supply, and food supply. Involvement of these sectors and the public in all stages of HIA provides stakeholders with the opportunity to engage with the activity and act collaboratively to share possible community benefits as well as to minimise potential future problems. The activities of these sectors impact on health and so need to be included in processes to determine risks and potential adaptation strategies.

## 4. Climate Change in Vanuatu

Vanuatu is an archipelago of approximately 80 islands with a land area of 12 335 square kilometres located south of the equator in the Western Pacific ocean. The predominantly Melanesian population of approximately 240 000 is growing at a rate of 2.3% per annum, and is expected to double by approximately 2030 (Ministry of Health Annual Report, 2010).

The economy is largely driven by tourism (which accounts for approximately 40% of Gross Domestic Product, (GDP)) and primary industries (agriculture, fisheries and forestry together account for roughly 15% of GDP).

Vanuatu’s climate is tropical, with two distinct seasons – a warm, wet season and a cooler, dry season. The climate varies considerably from year to year, mainly due to the effects of the El Niño-Southern Oscillation (ENSO) system. The wet season often brings tropical cyclones: 94 intense storms passed within 400km of Port Vila from 1969 to 2010 (Pacific Climate Change Science Program, PCCSPPCCSP, 2011).

The main climate change phenomena expected to occur in Vanuatu include (PCCSP, 2011):

- *increasing air and sea-surface temperatures*
  ○ average air temperatures in Vanuatu are expected to increase by up to 1°C by 2030 and in the order of 2-3°C by 2090, depending on future greenhouse gas emissions scenarios.
- *altered rainfall patterns*
  ○ most models predict drier dry seasons and wetter wet seasons for Vanuatu, as well as more “extreme/high” rainfall events.
- *less frequent but more intense cyclones*
- *sea-level rise*
  ○ the recent rate of sea-level rise in Vanuatu has been between 4.7 and 6 millimetres per year and is expected to continue at this rate to 2030.
- *ocean acidification*

### 4.1 The Adaptation Project

The objectives of this research were to:

- identify the potential risks to health from climate change in Vanuatu;
- evaluate those risks to determine their respective priorities in terms of the likelihood of the event occurring and the severity of the potential impact on human health and safety; and
- propose a range of feasible adaptation options to avoid the most serious impacts of climate change on health in Vanuatu.

## 5. Methods

The HIA framework used incorporated the profiling to risk management components. The research was guided by a sequence of three steps: planning, implementation and development of adaptation strategies.

For this project, health was considered in broad terms with a range of determinants, as per the WHO definition of environmental health:

*“Environmental health addresses all the physical, chemical, and biological factors external to a person, and all the related factors impacting behaviours. It encompasses the assessment and control of those environmental factors that can potentially affect health. It is targeted towards preventing disease and creating health-supportive environments” (WHO, 2013a)*.

Participants were invited to participate based on their knowledge, expertise and access to data and information relevant to Vanuatu in the areas of:

- The bio-physical environment (water, air quality, ecosystems)
- The social and economic environment (e.g economy, mental health, communities and lifestyle, dislocation)
- The built environment and infrastructure (transport, energy, essential services)
- Environmental diseases (vectors, pests, communicable diseases)
- Food security and safety
- Disaster and management (extreme events)
- Risk assessment and management

An emphasis was placed on the inclusion of community participants with understandings of local circumstances and variability.

### 5.1 Planning

An inception process with representatives of several government sectors detailed a stepwise approach to enable systematic progress through each stage. Table 1 provides a summary of the steps in the process.

**Table 1:** Summary of the climate change and health assessment process

Stage	Process Component	Issues for inclusion
Preliminary	Development of Communication Strategy Development of a Stakeholder involvement Strategy	
1	Identification of climate variables	Creation of scenario for 2030
2	Identification of environmental impacts arising from climate change	Addressed through identification of changes to: Biophysical environment
		Social environments
		Infrastructure
3	Identification of potential health impacts	Identification of health impacts arising from environmental changes
		Identification of vulnerable:
		People/groups
		Regions
		Infrastructure
		Services
		Identifying/determining gaps in knowledge
		Understanding current coping (controls) capacity and limitations
4	Risk Assessment	Undertake risk assessments of the identified health impacts
		Identifying experts to assist:
		Risk assessments
		Specific fields
5	Risk prioritisation	List impacts according to level of risk
		Need to reach consensus based on expert knowledge
6	Development of adaptation responses	Use of range of adaptation responses provided.
		Should consider other adaptations applicable to home country
		Consideration of responses with respect to:
		General population
		Vulnerabilities
		Adequacy of control measures
		Other requirements
		Priorities for action
7	Action Plans (strategies required to implement adaptations)	For application in Vanuatu
		Need to identify roles and responsibilities

The research process generally followed the steps of a climate change and health vulnerability and adaptation assessment (Spickett et al., 2011) and was divided into the phases of:

- Identification of the potential health impacts of climate change;
- Stratification (ranking) of the climate-sensitive health issues according the risk each posed in the context of climate change;
- Identification of appropriate adaptation strategies to reduce the risks posed by each climate-sensitive health issue

### 5.2 Implementation

Focus group meetings were held with stakeholders from relevant sectors whose activities were identified as potentially influencing the health impacts from climate change and included senior representatives from most Government sectors responsible for policy development and implementation.

To identify potential health impacts the groups were provided with a scenario of potential changes in climate in Vanuatu in the year 2030 based on projections from PCCSP (Pacific Climate Change Science Program, 2011). Two important assumptions were then made for the entire project which were:

1) The year is 2030 and climate change projections have occurred
2) Only current management strategies for each health impact are taken into account

The group members then considered in the context of the determinants of health (WHO, 2013b), four major areas:

- Biophysical environment – impacts including water quality, air quality and biodiversity.
- Social environment - impacts including population displacement and mental health issues.
- Built environment - impacts related to services, infrastructure and economics, including resource availability and access to a range of health, emergency and other services.
- Environmental diseases and food – impacts related to production of food, vector-borne and food-borne disease and other environmental diseases.

The climate change effects were also divided into broad sections:

- Increase in severity &/or incidence of extreme events (tropical cyclones, storms, droughts and heatwaves)
- Increase in temperature
- Changes in rainfall (patterns and volume)
- Increase in sea-level

For each potential climate change the group then identified:

- potential impacts on health
- potential health impact pathways
- vulnerable groups
- current responses and limitations
- gaps in knowledge

A comparative measure of risk is essential for the prioritisation of adaptation measures. Participants with expertise in health or risk assessment undertook a qualitative risk assessment in their specific areas of knowledge to ascertain the level of risk to public health in Vanuatu.

The potential impacts were divided into the areas of:

- Extreme Events
- Temperature Increase and Related Changes
- Water-borne Disease and Water Quality
- Vector-borne diseases
- Air Quality
- Food-borne diseases
- Food Production
- Social Impact/Community Lifestyle-Dislocation, Mental Health

Impacts were assessed on a qualitative scale that considered the health consequences and the likelihood of the health impact occurring. The consequences of potential health impacts were considered in terms of the magnitude of the impact, the severity of the health impact, the number of people affected, the duration of the impact and the socio-economic implications. Likelihood ratings were rare, unlikely, possible, likely and almost certain. A rationale for rankings was recorded.

Risk assessment results were entered into a risk assessment matrix to assign each identified health impact a risk category of low, medium, high or extreme. Risk priority levels determined by each group were compared to improve parity across differing impacts types. Consensus regarding the final risk level was important to enable focus on high-level risks for the development of potential adaptation strategies.

The risk management stage of the project considered adaptation measures that could be applied to the potential health impacts with a risk ranking of medium or higher. A literature search had identified a list of potential adaptation measures and participants considered each measure for Vanuatu and added other measures where appropriate.

### 5.3 Adaptation Strategies

The adaptation measures were categorised as:

- Legislative or Regulatory
- Public Education or Communication
- Surveillance and Monitoring
- Ecosystem Intervention
- Infrastructure Development
- Technological/Engineering
- Medical Intervention
- Research/Further Information

Each adaptation measure was considered in the context of:

- Relevance for Vanuatu
- Current capacity inclusive of vulnerable groups/regions rated as; not in place (N); inadequate (I); being developed (D); or adequate (A)
- How adaptations could be implemented in Vanuatu (adjustment/modification of existing measures or the development of new measures)
- Identification of sectors that would be involved in the development and implementation of the adaptation strategies.

## 6. Results

The application of an HIA framework addressing climate change in Vanuatu provided:

- Identification of potential health impacts
- Identification of vulnerable groups
- Understanding of key current controls or coping strategies
- Determination of current knowledge and gaps
- Identification of linkages between sectors
- Assessment of risk associated with each impact
- Identification of opportunities for adaptation and responsible sectors

Health problems that may be affected by climate change in Vanuatu were identified. These include (but are not limited to):

- vector-borne diseases (eg. malaria, dengue fever, lymphatic filariasis)
- respiratory disease
- water-borne diseases
- malnutrition/food security
- food-borne diseases
- non-communicable diseases
- traumatic injuries and deaths (eg. from extreme weather events such as cyclones, floods)
- temperature-related illnesses
- mental health disorders
- skin conditions
- eye diseases

Based on feedback from expert participants, which included relevant evidential information and data, climate-sensitive health risks in Vanuatu were ranked as per the results in Table 2.

**Table 2:** Climate-sensitive health risks in Vanuatu

Risk category	Health issue
Extreme	Water-borne diseases
	Food-borne diseases
High	Vector-borne diseases
	Malnutrition
	Non-communicable diseases
	Temperature-related illnesses
	Occupation-related illnesses
Medium	Respiratory infections
	Skin conditions
	Eye diseases
	Mental health disorders
	Traumatic injuries and deaths

The highest level of risk was assigned to health impacts from water-borne and food-borne diseases.

Common themes of vulnerability across a wide range of health impacts were considered under the categories; regional, economic, social and infrastructure and services. These highlighted that existing health vulnerabilities are likely to be exacerbated by climate change.

Table 3 lists potential adaptation strategies proposed to manage the climate-sensitive health risks for the extreme risk categories. Adaptation strategies were also developed for the health risks in the high category but they are not presented here.

**Table 3:** Potential adaptation strategies and actions for priority climate-sensitive health risks in Vanuatu

Strategies	Actions
**EXTREME RISK – Water Borne Diseases**	
Legislative or Regulatory	- Develop policy for water storage design and maintenance
	- Review, amend and enforce existing relevant legislation such as the Public Health Act and Water Resource Management Act
	- Finalize and enforce policies and standards for water management
	- Fast track the completion of the National Building Code and mainstream Climate Change and Water considerations
	- Implementation of National Water Strategy 2008-2018.
Public Education & Communication	- Develop water hygiene communication strategy
	- Strengthen community participation in health promotion activities
	- Mainstream climate change and water hygiene into national curricula for schools and other educational programs at al levels
Surveillance & Monitoring	- Strengthen water and waste water quality monitoring
	- Strengthen water quantity monitoring
Ecosystem Intervention	- Identify and map water catchment areas
	- Develop water shed management plans
	- Protect Water Source areas/Catchment areas.
Infrastructure Development	- Strengthen and expand National Water Laboratory capacity
	- Establish and/or upgrade public waste water treatment plant in major urban centres
	- Increase and expand distribution of health facilities to remote areas
Technology or Engineering	- Improve storm water drainage systems
	- Climate proof designs for public facilities
	- Use renewable energy technologies to power health facilities
	- Establish appropriate waste management processes
	- Establish and manage stock pile of medical & water storage supplies for national health response
Health Intervention	- Mainstream climate change and water hygiene into National Health Disaster Plan
	- Deployment of more doctors to rural health centres
Research/Information	- Assessments of water and climate change issues and identification of vulnerable communities
	- Establish relationship between ENSO, temperature/precipitation and incidences of water borne diseases
	- Strengthen and update Health Information Systems – particularly water hygiene and Environmental Health diseases
	- Complete national water resource inventory
	- Conduct national environmental health survey
Capacity Building	-Establish and implement national strategy for water hygiene Strengthen capacity to develop and implement education and training curricula

**EXTREME RISK – Food Borne Diseases**	
S1 Legislative or Regulatory	- Review, Amend and Enforce Food Control Act and regulations
	- Development of National Food Standards
	- Develop National Food Security Framework
S2 Public Education & Communication	- Develop Food Safety Communication strategy
	- Mainstream climate change and food safety into National curricula
	- Strengthen health promotion at all levels
Surveillance & Monitoring	- Develop surveillance system for food borne disease/health information
	- Strengthen existing laboratory capacities
	- Establish Food Import/Export Inspection Systems.
Ecosystem Intervention	- Protect Fishing grounds/Agricultural land
	- Establish aquaculture
Infrastructure Development	- Establish National Food Analytical facilities.
	- Improve food storage, transport and marketing facilities to remote areas
	- Improve food-processing facilities.
	- Provide assistance to street vendors in establishing safe areas for handling food.
Technology or Engineering	- Develop and implement Good Agriculture Practices
	- Provide support for local and traditional processes and practices
	- Introduce renewable energy technologies for food processing
Health Intervention	- Review and update the National Health Workers manual on treatment of Diarrheal diseases and other dehydration conditions.
	- Deployment of more doctors to rural health centres
Research/Information	- Strengthen existing data collection procedures under the Health Information System
	- Establish links between climate parameters and food poisoning/intoxication and specific pathogens and toxins
	- Research traditional treatments for Fish Poisoning
	- Conduct a National Food Safety/Nutrition Survey.
Capacity Building	- Strengthen and enhance enforcement/inspection capacities
	- Increase Human resource capacities in the Environmental Health Unit.

## 7. Discussion

The main potential health impacts from climate change in Vanuatu tended to emphasise the public health risks that are dominant in a society experiencing the so-called “epidemiological transition”, with relatively high burdens of both infectious and non-communicable diseases. It is important to note that, in the case of Vanuatu as in many other countries and communities, climate change will not necessarily bring new threats, but rather act as an “amplifier” or “multiplier” of existing health problems (that is, in the absence of effective adaptation strategies).

A major difficulty was dealing with the significant uncertainties. Typical quantitative risk assessment procedures rely on well-documented risks and, with the availability of adequate data reasonably good risk estimates can be calculated. Making judgements about risks to human health is more difficult because of the uncertainty from interacting climatic variations and consequential environmental changes. In addition, there were uncertainties about the proposed adaptations as workshop participants were not fully aware of the status of the current circumstances of the proposed adaptation measures for reducing health impacts in Vanuatu.

The levels of uncertainty surrounding consequences and/or likelihood of the potential health impacts were typically higher for indirect and social health impacts, often because of the complexity of the relationship between the climate variable and the health impact, and knowledge gaps about this relationship. The vulnerabilities of different groups within the population were also considered. In general a higher level of conservatism was applied for those health impacts with a high degree of uncertainty.

The levels of urgency considered necessary to address the climate-sensitive health issues and progress adaptation options are summarised in Table 4.

**Table 4:** Management of climate-sensitive health risks

Risk Levels for Health	Description of Management Action
Extreme	Risks require urgent attention at the most senior level and cannot be simply accepted by the community
High	Risks are the most severe that can be accepted by the community and need planned action
Medium	Risks can be expected to be part of normal circumstances but maintained under review by appropriate sectors
Low	Risks will be maintained under review but it is expected that existing controls will be sufficient and no further action will be required to treat them unless they become more severe

In most, if not all, of the adaptation options listed, there are common actions that include the need for:

- increased capacity both in human resources and equipment and other support;
- further information on the health impacts of climate change, including incorporation of these considerations into the training curricula of health professionals in Vanuatu;
- community education from primary school onwards on the potential health impacts of climate change and the need for adaptation strategies;
- improved collection, collation, storage and analysis of data on health status in the community;
- inter-sectoral collaboration; and
- improved standards and better enforcement of current regulations.

It was recommended that the adaptations for the “extreme” and “high” risk categories be given priority consideration through a whole-of-government approach. The next stages would be to implement the adaptation measures in each area via a lead agency or sector together with other relevant sectors. The health sector should be included in all groups including anticipation of any unexpected or unforeseen adverse health impacts. The Health Impact Assessment (HIA) process would provide a framework for this process to occur.

Responses should determine whether each of the proposed adaptations require further justification and can be implemented readily or if further analysis is required to evaluate the nature of the risk and determine the most appropriate response actions. Some risks may need to be accepted if there is no cost-effective adaptation measure or the risk is considered insignificant.

The process used in this project should be repeated, in a modified form, as new information on the monitoring of climates parameters, predicted climate changes and the predicted adverse impacts on human health become available.

The limitations of this project have been recognised. Fussell (2008) notes that there are many aspects of climate change impacts that have unfamiliar components such as the spatial scale, its long-term horizon and its complex spatial and time pattern. The use of a conservative scenario for 2030 was considered most appropriate for this investigation. For a more rigorous assessment of the potential health impacts of climate change, there is a need to ensure that outcomes are reassessed as climate predictions change, downscaled climate data for specific regions are developed and utilised and that all potential affected sectors are informed and consulted during all stages.

In considering the adaptation activities it is important that, for every action, the potential co-benefits (for health) are also considered.

## 8. Conclusions

This research has identified many potential adaptation measures to reduce or mitigate the impact of climate change on human health in Vanuatu that consider the current level of development in the country. The possible events that could impact on health have been identified in terms of the estimated level of risk and the estimated current level of capacity response. This assessment should allow sectors to make judgements about risks and appropriate responses that require attention in the short term, those that can be set aside for later attention and those where more information is needed. The results are expected to be used by decision makers to provide direction on planning for the short, medium and long term.

In the final analysis it may be that some risks will need to be accepted because there is no cost effective adaptation measure or the risk to human health is considered to be insignificant in Vanuatu. The level of risk assessment used in this project did not require a detailed understanding of climate change to provide a general indication of the types of adaptation responses needed to reduce the adverse effects on health which may arise. However, further information is needed in order to progress to a more detailed and accurate assessment of current adaptation measures. The activities and requirements of specific sectors will need a greater level of general awareness and increased capacity to more accurately predict the impacts of climate changes on health and to develop and implement further effective adaptation strategies.

Additionally there needs to be improvement in environmental and health monitoring and surveillance systems across Vanuatu. The health care sector could provide low cost monitoring mechanisms for specific vulnerable groups and hence sentinel data.

Although this project has identified many potential adaptation responses for Vanuatu, relatively little is known about the potential barriers to and opportunities for the introduction of the strategies and their cost effectiveness. Thus there is the need for more investigation/research into these issues. These processes also need to be linked with climate change activities by other organizations.

Recent increased awareness of changes in the climate and the potential impacts this may have on our health and way of life have resulted in an increased interest and concern about mitigation of adverse effects and implementation of adaptation measures to reduce adverse impacts. As more information becomes available from scientists and other specialists, it is clear that adaptation strategies need to be formulated for all sectors including health.

The extent of impacts from adverse effects will depend on how well society in Vanuatu can estimate the level of the impacts, the planning processes for adaptation strategies and the successful implementation of the adaptation measures. Concurrent with these processes will be measures to mitigate the changes in the various climatic parameters, which can result in environmental impacts.

It is accepted that climatic conditions in Vanuatu are changing and that physical and environmental changes will influence the way the community lives. This project has identified a number of potential health impacts that may arise from climate change in Vanuatu, and has considered a range of ways in which these could be managed. A number of potential adaptations that could be implemented across Vanuatu to avoid some of the more serious impacts of climate change on health have been identified and a model procedure which can be used, with some modification, to develop revised adaptation strategies as the predicted climate variables change and adaptation strategies has been introduced.
